# Catamenial Pneumothorax—Still an Unveiled Disease

**DOI:** 10.3390/medicina60122029

**Published:** 2024-12-09

**Authors:** Iwona Damps-Konstańska, Adriana Szukalska, Piotr Janowiak, Ewa Jassem

**Affiliations:** 1 Department of Pneumonology, Medical University of Gdańsk, 80-210 Gdańsk, Poland; iwona.damps-konstanska@gumed.edu.pl (I.D.-K.);; 2 Department of Hematology, University Hospital No. 2 Dr. Jan Biziel in Bydgoszcz, 85-168 Bydgoszcz, Poland

**Keywords:** pneumothorax, catamenial pneumothorax, endometriosis

## Abstract

This review presents current opinions on an uncommon condition called catamenial pneumothorax (CP), which is usually associated with thoracic endometriosis syndrome (TES). TES is characterized by the presence of endometriotic lesions in pleura and lung parenchyma and presents with various clinical signs and symptoms, including catamenial pneumothorax. Their diagnosis is often delayed. Pulmonary endometric lesions, however, often detected in patients with hemothorax and hemoptysis, may be absent in a proportion of cases of pneumothorax. The typical presentation of CP includes signs and symptoms of pneumothorax, which occur along with menstruation, most commonly around 24 h before and 48–72 h after its onset. However, they may not occur during every menstrual cycle. Suggestive CP lesions on conventional radiography (RTG) include pneumoperitoneum accompanying right-sided pneumothorax, lung opacities, pleural effusion, and nodular infiltrates. Chest and abdomen computed tomography (CT), particularly contrast-enhanced, may additionally show pneumoperitoneum and diaphragmatic lesions. The management of CP includes supportive treatment of acute symptoms and causal treatment to prevent recurrent disease. This article presents the pathophysiology of CP, an overview of the diagnostic methods, and the current therapeutic approaches. The necessity for a multidisciplinary approach to the diagnosis of CP and to the choice of the best treatment modality is underlined (promising new therapeutic options are also mentioned); however, international guidelines are still missing.

## 1. Introduction

Catamenial pneumothorax (CP) is an uncommon condition, usually associated with thoracic endometriosis syndrome (TES) [1]. TES is characterized by the presence of endometriotic lesions in pleura and lung parenchyma and presents with various clinical signs and symptoms, including catamenial pneumothorax (30–73%), catamenial hemothorax (14%), catamenial hemoptysis (7%), and lung nodules (6%) [2]. Although endometriosis is known to affect around 3–10% of women of childbearing age and approximately 2–5% of women after menopause, TES and CP remain a rare condition. Their diagnosis is often delayed [3]. Pulmonary endometric lesions, however, often detected in patients with hemothorax and hemoptysis, may be absent in a proportion of cases of pneumothorax. This phenomenon may indicate a different pathophysiological mechanism of CP. Usually, surgery is the first intervention, but most patients will require additional treatment.

This article reviews the diagnostic and therapeutic options available for CP.

## 2. Pathogenesis

Maurer et al.’s first disease report comes from 1958 [4]. The name “catamenial” derives from the Greek word “katamenios” and translates into “monthly occurrence” [5]. It is widely accepted that endometriotic lesions in pleura and lung parenchyma are responsible for CP in a proportion of patients. However, the exact mechanism of their occurrence is not well recognized. Furthermore, for the cases in which endometriotic lesions cannot be found, other pathophysiology has been proposed to explain CP (Table 1).

### 2.1. Theories for the Development of Endometrial Foci

Various theories have attempted to explain the mechanism of extrapelvic endometrial tissue development. According to the oldest theory of “retrograde menstruation”, dating back to 1927, menstrual blood flows from the uterus through the fallopian tubes into the pelvis and further to the abdomen. Endometrial cells’ repetitive proliferation and necrosis around the diaphragm area leads to diaphragmatic fenestration formation [6]. As a result, endometrial cells pass into the chest and visceral pleura, causing alveolar injury and pneumothorax [1]. The second, metastatic theory, suggests that endometrial cells spread from the uterus via lymph or blood vessels—their proliferation and necrosis in the pleural space results in pneumothorax [9]. In turn, the vascular embolization hypothesis postulates that fragments of the endometrial tissue travel in the venous system from the uterus into the right heart and eventually deposit in the lung parenchyma and pleura [10]. Another assumption states that the microvascular endothelium of ectopic endometrial tissue derives from endothelial progenitor cells, which develop de novo in vasculogenesis, as opposed to traditional angiogenesis [11].

Several studies demonstrated that autoimmune diseases are more common in women with endometriosis, which suggests that systemic immune alternations may contribute to the disease pathogenesis [12]. Patients with endometriosis present with elevated concentrations of activated macrophages and decreased cytotoxic T-cell and natural killer cell activity [13,14]. A possible explanation for the development of thoracic foci could be the regurgitation of endometrial debris into the lung parenchyma (retrograde menstruation), followed by a defective “immune surveillance” reaction [13]. Alternatively, it may result from defective cytotoxic natural killer cell activity and the inability to eliminate ectopic endometrial cells [15]. Additionally, cytokines and growth factors secreted by ectopic endometrial cells may be responsible for the activation of cell proliferation and angiogenesis, consequently producing thoracic lesions [13].

### 2.2. Theories for the Development of CP Without Endometrial Foci

In some cases, a lack of intrathoracic endometriotic lesions may be caused by air passing from the uterus into the fallopian tubes, which results in increased permeability of menstruation blood to the peritoneal cavity and via diaphragmatic fenestrations into the pleural space. It is also believed that high F2 prostaglandin levels during menstruation induce vasoconstriction and bronchospasm, resulting in alveolar rupture and pneumothorax [8].

## 3. Clinical Presentation

The typical presentation of CP includes signs and symptoms of pneumothorax, which occur along with menstruation, most commonly around 24 h before and 48–72 h after its onset. However, they may not occur during every menstrual cycle [16,17]. The disease typically affects women of reproductive age, less frequently young girls and post-climacteric women [18]. CP most commonly develops on the right side (85–95%) due to the clockwise circulation of peritoneal fluid [1,16].

The clinical manifestations of CP may include chest pain, shortness of breath, increased heart rate, rapid breathing, cough, fatigue, and pleural effusion. A history of infertility, chronic pelvic pain, and dysmenorrhea or dyspareunia may be suggestive of TES. In some cases, endometriosis-related diaphragmatic hernia may be present [19,20]. Other symptoms can vary depending on the foci location. Those located more centrally usually cause hemoptysis [21]. Occasional symptoms also include hemothorax and chest or scapular pain [5,22]. There was also a case of pulmonary endometriosis mimicking an acute abdomen [23,24]. Symptoms’ intensity exacerbates around the time of menstruation and may vary from very mild to severe. Significantly, pneumothorax may be associated with decreased lung function and should be treated promptly. The development of tension pneumothorax could potentially be fatal; therefore, in some cases, a chest tube insertion may be necessary to allow for re-expansion of the lung tissue.

A comprehensive clinical examination and medical interview, including family gynecological history, are necessary to facilitate proper diagnosis [1,2]. The reoccurrence of characteristic symptoms during menstruation in reproductive-age women should hint at the possibility of CP. An elevated level of CA-125 may accompany the disease. Although this biomarker is not specific, it can support early diagnosis of CP related to thoracic endometriosis [25]. Some studies suggest that elevated CA-125 could help implement prompt therapy to prevent reoccurrence of CP [26]. However, this approach warrants prospective evaluation in a large-scale study. Some putative biomarkers related to nerve fiber growth or cell cycle control are promising candidates for allowing a non-invasive CP diagnosis [27].

## 4. Diagnostic Methods

Suggestive CP lesions on conventional radiography (RTG) include pneumoperitoneum accompanying right-sided pneumothorax, lung opacities, pleural effusion, and nodular infiltrates [28,29]. In some cases, extensive diaphragmatic features and multiple “air-filled bubbles”—small diaphragmatic perforations—may also be present [30]. Chest and abdomen computed tomography (CT), particularly contrast-enhanced, may additionally show pneumoperitoneum and diaphragmatic lesions [31,32]. The CT should preferably be performed during menstruation [33]. High-resolution CT is preferred, as it best determines the anatomical localization of ectopic plaque before thoracic surgery. It is also important to carefully screen the posterosuperior diaphragm, a relatively common disease location [33].

Although commonly used, both RTG and CT have poorer specificity in CP than magnetic resonance imaging (MRI) in detecting endometriosis-related hemorrhage. Like RTG and CT, the optimal time to perform MRI is during menstruation. MRI has lower spatial resolution than CT but provides better contrast resolution and more precise characterization of hemorrhagic lesions [34]. CP lesions have various features, including nodules, opacities, bullous formation, and ground glass infiltrates [35]. A study by Rousset et al. showed that MRI allows for identifying diaphragmatic nodules with up to 83% sensitivity [35]. Fat-suppressed T1-weighted sequences are optimal for detailed examination. Most lesions appear right-sided and are hyperintense.

Bronchoscopy is not routinely recommended since most lesions occur in the peripheral lung parenchyma [26,36]. However, bronchoscopy may be helpful in selected cases if performed during the first two days of menstruation [37]. On the contrary, video-assisted thoracoscopy (VAT) exploration of the thoracic cavity around the menstruation time allows for optimal visualization of ectopic lesions [6,38]. Extensive lung, visceral, parietal pleurae, and pericardium examinations seem compulsory. Any bullae, air leaks, or blebs must be identified. Care should be taken when inspecting the diaphragm, especially the posterosuperior surface, as thoracic lesions are commonly found in this area. However, classic thoracotomy has been replaced by thoracoscopy/VATS due to its less invasive nature, less pain, faster patient recovery, and better cosmetic results in young women. A video-assisted mini-thoracotomy is recommended instead of VATS when multiple diaphragmatic lesions are present within the diaphragm or in individual cases where endoscopy is unsafe. Otherwise, VATS is considered superior to classical thoracotomy due to better magnification, allowing for the detection of the smallest endometrial foci [34]. Histopathological tissue examination is essential to confirm recurrent disease.

There are no specific pathological diagnostic criteria for CP. The final confirmation is established by histopathological examination of the tissue material acquired via VATS or open thoracotomy. The typical features of CP include the presence of endometrial stroma, glans, and hemosiderin-filled macrophages [16]. Immunostaining for estrogen and progesterone receptors was postulated to support the diagnosis [39,40]. Furthermore, circulating endometrial cells (CECs) were demonstrated in the blood of CP patients [41]. They may present four phonotypes: epithelial, stem cell-like, stroma-like, and glandular. It was postulated that CECs can be used to distinguish between catamenial and spontaneous pneumothorax [42].

Up to 80% of women with thoracic endometriosis have co-existing abdominopelvic endometriosis [43]. Ultrasonography may be used to detect abdominal or pelvic endometriotic lesions, which are usually hypoechoic with internal vascularity and cysts [44]. It can also detect endometriosis-related peritoneal fluid and monitor CP.

**Figure 1:**
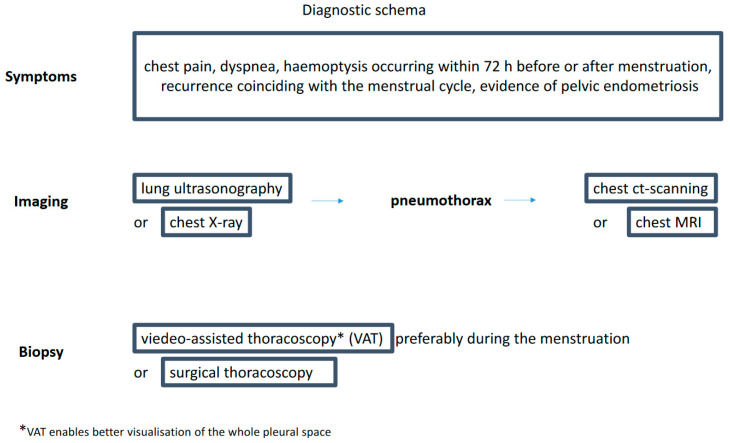
Diagnostic schema.

The long list of available diagnostic tests may give the impression that diagnosing CP is simple (Figure 1). However, a recent survey among senior members of the British Society for Gynecological Endoscopy showed the lack of common diagnostic and therapeutic CP pathways in the United Kingdom [45]. There is no diagnostic algorithm for this type of pneumothorax proposed in the guidelines by the BTS Pleural Disease Guideline Group [46]. 

## 5. Treatment

**Figure 2:**
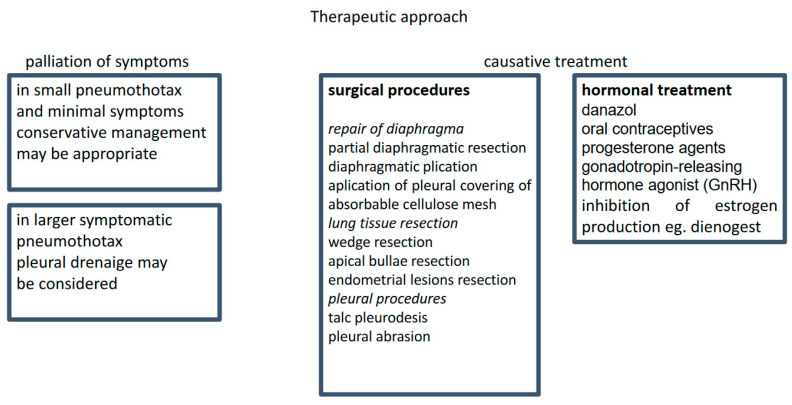
Treatment strategy.

The management of CP includes supportive treatment of acute symptoms and causal treatment to prevent recurrent disease (Figure 2). There is no convincing evidence regarding the optimal supportive care. A meta-analysis including 6344 and 5578 patients treated with tube drainage or conservative management, respectively, showed no significant differences in recurrence rates between these two approaches [47].

There is a selection of surgical and pharmacological therapeutic options. However, there are no guidelines defining the right choice for the individual patient.

Casual treatment includes thoracic surgery and hormonal therapy, a combination that is considered the most effective [48,49]. However, in the study of Kim et al., of 27 patients treated with hormonal therapy, 8 experienced a recurrence within 1 year, and the diaphragm resection was the only independent protective factor [50]. Thoracic surgery for CP is relatively safe and has almost no mortality. However, a 4-year recurrence rate is in the range of 8–40%. In the study of Joseph et al., the recurrence rate for patients who did and did not receive postoperative hormonal treatment was 60% and 30%, respectively [1]. The management is difficult because of the high recurrence rate. In women who underwent VATS, the recurrence rates in CP, non-CP but endometriosis-related, and non-CP non-endometriosis-related pneumothoraces were 32, 27, and 5.3%, respectively [7].

### 5.1. Surgery

Surgical treatments are nowadays performed via VATS. The most frequent surgical procedures include bullectomy, pleurectomy, or pleurodesis. In a review study including 195 CP patients, 154 (79%) were treated surgically; of those, 33% underwent pleurodesis, 39% diaphragmatic repair, and 20% lung wedge resection [6]. Two types of pleurodesis were used: mechanical (achieved by abrasion) and chemical (usually performed with the use of talc). The mean relapse-free time after diaphragm excision (with or without pleurodesis) or pleurodesis alone was 24 months and 61 months, respectively [44]. However, these results are likely to be associated with diaphragmatic defects rather than with the extent of surgery. The lining of the diaphragmatic surface with a polyglactin mesh, polypropylene polytetrafluoroethylene mesh, or bovine pericardial patch (after cessation of hormonal treatment) provides additional support for the diaphragm, close diaphragmatic perforations, and induce fibrotic adhesion to the surrounding lung tissue [51,52]. These methods allow for durable freedom from recurrence and have been proposed for all patients, as endometriosis and diaphragmatic defects may not be evident during surgery [53]. Interestingly, endometriotic pleural nodules were more frequently associated with inferior outcomes (prolonged air leak and early recurrence) than diaphragmic defects [54].

Generally, pleurodesis is a procedure considered safe in the short- and long-term observation [55,56]. Some authors state that pleural abrasion alone is sufficient to control CP symptoms, whereas others suggest removing all visible lesions to prevent further intrathoracic dissemination. According to the lesion location, their recommendations include resection of endometriotic lesions or bullae, partial diaphragm resection of parietal pleura, or limited lung wedge resection [57,58]. Due to the high disease recurrence rates after surgical treatment, they also agree on placing polyglactin mesh over the diaphragmatic surface [59].

### 5.2. Hormonal Therapy

Pharmacotherapy aims to suppress ovarian estrogen production and atrophy of functional endometrium (including ectopic endometrium within the chest cavity), resulting in a lack of menstruation. Commonly used pharmaceutics include danazol, oral contraceptives, progesterone agents, and gonadotropin-releasing hormone (GnRH) agonists [1,51,60].

There is a general agreement that hormonal treatment supports the prevention of recurrent disease and is particularly useful in high-risk patients [1]. The use of GnRH analogs has been correlated with a lower incidence of CP recurrence than surgery alone or postoperative estrogen-progesterone therapy. Several authors have demonstrated a high efficacy of GnRH in preventing CP recurrence [61]. Hormonal treatment should be commenced immediately after the surgery to suppress ectopic endometrial tissue activity and allow enough time for the pleural adhesions to mature [59,62]. Cyclic hormonal changes could disrupt this process, reduce adhesion formation, and increase the risk of disease reoccurrence. Another option is a short treatment with GnRH agonist before and shortly after the surgical treatment to allow for maturation of the pleurodesis [59].

It is necessary to consider the patient’s plans regarding pregnancy before treatment induction. Various side effects, such as tiredness, digestive system problems, weight gain, hair thinning, and depression, may develop during GnRH therapy; therefore, it must be time-limited [50]. Treatment tolerance and patient preference are essential aspects that have to be considered to obtain satisfactory results.

## 6. Conclusions

**Figure 3:**
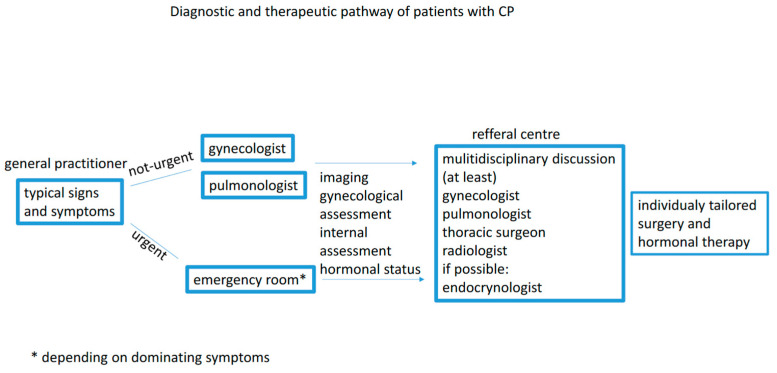
Diagnostic and therapeutic pathway.

The awareness of cyclic reoccurrence of severe symptoms around the time of menstruation may significantly reduce the quality of life and prevent life-threatening complications. The guidelines on diagnosing and treating CP are still lacking. Hence, a modern approach should include a medical pathway for each patient and should be developed by a multidisciplinary board involving a pulmonologist, gynecologist, surgeon, and radiologist (Figure 3).

### Future Directions

Recently, an improvement in the understanding of the endometriosis angiogenic network regulation system has brought new hope for its diagnosis and treatment. Currently, the primary anti-angiogenic drugs used in the targeted therapy include anti-VEGF antibodies (bevacizumab, ranibizumab), VEGFR tyrosine kinase inhibitor (sorafenib, sunitinib), COX-2 inhibitor (celecoxib, parecoxib), dopamine receptor agonists (cabergoline), and others [63,64,65,66,67,68,69]. The angiotensin II receptor blocker, telmisartan, effectively inhibits vascularization and the growth of murine endometriosis-like lesions [70]. The most promising of these potential therapies are currently being investigated for further testing in both rodent and nonhuman primate trials.

Future research should focus on standardizing surgical management according to the type of pleural lesion. Multicenter studies, including larger series of patients and new techniques, such as confocal microscopy or AI, may refine treatment recommendations. The same holds true for hormonal treatment—larger studies are necessary to define its optimal duration and most effective drugs.

## Figures and Tables

**Table 1:** Theories for development of endometrial foci.

Retrograde menstruation [1,6]	Migration of endometrial debris from the uterus through the fallopian tubes into the lesser pelvis and further. Repetitive proliferation and necrosis of endometrial cells around the diaphragm area leads to the formation of diaphragmatic fenestrations. Passage of endometrial cells through the fenestrations into the chest and visceral pleura leads to alveolar damage and pneumothorax.
Transdiaphragmatic air passage [7]	The passage of air from the uterus via the diaphragmatic fenestrations into the pleural cavity results in the development of pneumothorax.
Physiological [8]	Increased prostaglandin F2 production during menses leads to vasoconstriction and bronchospasm, resulting in alveolar rupture and pneumothorax.
Metastatic [9]	Lymphatic or hematogenous spread of endometrial cells from the uterus into extrapelvic localisations.
Coelomic metaplasia [1,9]	Metaplasia of mesothelial cells that line the thoracic cavity.
Vascular embolization [10]	The endometrial debris travels in the venous system from the uterus to the right heart and into the pulmonary circulation, where it is deposited in the lung pleura or parenchyma.
Vasculogenesis [11]	De novo development of microvascular endothelium of ectopic endometrial tissue from endothelial progenitor cells.
Immune dysfunction [12,13,14]	Defective “immune-surveillance”added reaction induced by the inflammatory response. Defective cytotoxic natural killer cell activity and inability to eliminate ectopic endometrial cells. Secretion of cytokines and growth factors by ectopic endometrial cells, which induce the development of thoracic lesions by activating cell proliferation and angiogenesis.
